# A Rare Case of ST-Segment Elevation Myocardial Infarction and Left Ventricular Thrombus in a Dextrocardia Patient With COVID-19 Infection

**DOI:** 10.7759/cureus.33527

**Published:** 2023-01-09

**Authors:** Nicholas Roma, Kashyap Shah, Bruce Ferraro, Matthew Durkin, Matthew Krinock, Michael Durkin

**Affiliations:** 1 Internal Medicine, St. Luke's University Health Network, Bethlehem, USA; 2 Cardiology, St. Luke's University Health Network, Bethlehem, USA; 3 Cardiology, St. Luke’s University Hospital, Bethlehem, USA; 4 Cardiology, St. Luke's University Hospital, Bethlehem, USA

**Keywords:** acute coronary syndrome, dextrocardia, thrombus, covid, stemi

## Abstract

Acute coronary syndrome (ACS) is an increasingly common finding among patients presenting with Coronavirus Disease 2019 (COVID-19) pneumonia. While cardiovascular disease alone remains one of the most common causes of death among COVID-19 patients in the United States, its heightened prevalence with COVID-19 pneumonia has been well documented. Here we present the case of a 58-year-old male with an extensive cardiac history including coronary artery disease (CAD) with multiple drug-eluting stents (DES) placed and an episode of cardiac arrest requiring implantable cardioverter defibrillator (ICD) placement. He presented to the Emergency Department originally complaining of chest pain, shortness of breath, and fatigue, and was found to be positive for COVID-19 pneumonia. Cardiac catheterization demonstrated extensive CAD and evaluation for coronary artery bypass grafting (CABG) was warranted. Shortly after, the patient experienced an acute thrombotic episode in the left anterior descending (LAD) coronary artery and underwent successful emergent high-risk percutaneous coronary intervention (PCI) with DES placement. The patient was also found to have a left ventricular thrombus requiring anticoagulation. Despite his complex course, the patient had a very favorable outcome.

## Introduction

Acute coronary syndrome (ACS) is a term used to describe the range of conditions associated with decreased coronary perfusion leading to myocardial ischemia. The term encompasses conditions such as unstable angina (UA), acute non-ST-elevation myocardial infarction (NSTEMI), and acute ST-elevation myocardial infarction (STEMI). The typical presentation includes severe chest pain or discomfort, along with other less specific symptoms such as dyspnea, diaphoresis, and nausea/vomiting. The prompt recognition of this condition through various modalities such as electrocardiogram (ECG) changes and troponin elevation is the key to successful management as treatment with percutaneous coronary intervention (PCI) has shown to be highly successful and is associated with mortality benefits [1]. This information is especially useful during the COVID-19 pandemic as the cardiovascular disease was shown to be the most prevalent medical condition in patients with COVID-19 with an association rate of 73.5% [2]. In addition, patients presenting with both COVID-19 and STEMI were found to have a higher coronary disease burden, including higher instances of in-stent thrombosis, multiple thrombotic lesions, and higher thrombus grades when compared to those without COVID-19 [3].

## Case presentation

A 58-year-old male presented with a chief complaint of chest pain and shortness of breath for the past three days. His past medical history is extremely complex including dextrocardia, atrial flutter, coronary artery disease (CAD), hypertension, and hyperlipidemia. The patient initially presented with cardiac symptoms several years ago and was found to have atrial flutter requiring ablation and then re-ablation years later. Additionally, he had a cardiac arrest requiring an implantable cardioverter-defibrillator (ICD) placement. After further workup of this cardiac arrest, the patient was found to have occlusions in the left anterior descending (LAD) 99%, distal right coronary artery (RCA) 50%, right posterior descending artery (PDA) 70%, and ostial marginal 1 (OM1) 90%. Drug-eluting stents (DES) were placed in the OM1 and LAD. Several months after his cardiac arrest and DES placement, he presented again with chest pain and shortness of breath. The workup included a repeat catheterization significant for occlusion of the proximal Ramus intermedius (RI) requiring another DES placement. The patient was still symptomatic with chest discomfort and shortness of breath specifically when exercising, so a repeat ischemic workup through nuclear stress testing was performed and no significant ischemia was found. The patient was managed medically at that time.

During his current encounter in the Emergency Department (ED), the patient presented with chest pain, shortness of breath, fatigue, myalgias, and fevers. He then tested positive for COVID-19. Of note, the patient was not vaccinated at the time. Initial workup included high-sensitivity troponin showed a large delta between the two and four hours (Table 1). Additionally, CRP, BNP, and D-dimer were elevated (Table 1). ACS protocol was started, and the patient was started on a Heparin drip, Aspirin 325 mg, Nitroglycerin 0.4 mg, Hydromorphone 0.5 mg, and continued home Atenolol 25 mg daily. Computed tomography angiography (CTA) was performed showing subsegmental right middle lobe pulmonary embolus as well as extensive bilateral ground-glass opacities consistent with COVID-19 pneumonia.

**Table 1:** Lab values on admission

	Value	Reference Range
Troponin (0 hour)	11 ng/L	<50 ng/L
Troponin (2 hour)	32 ng/L	<50 ng/L
Troponin (4 hour)	3530 ng/L	<50 ng/L
CRP	220 mg/L	<10.0 mg/L
BNP	304 pg/mL	<450 pg/mL
D-Dimer	>20 µg/mL	<0.50 µg/mL

**Figure 1:**
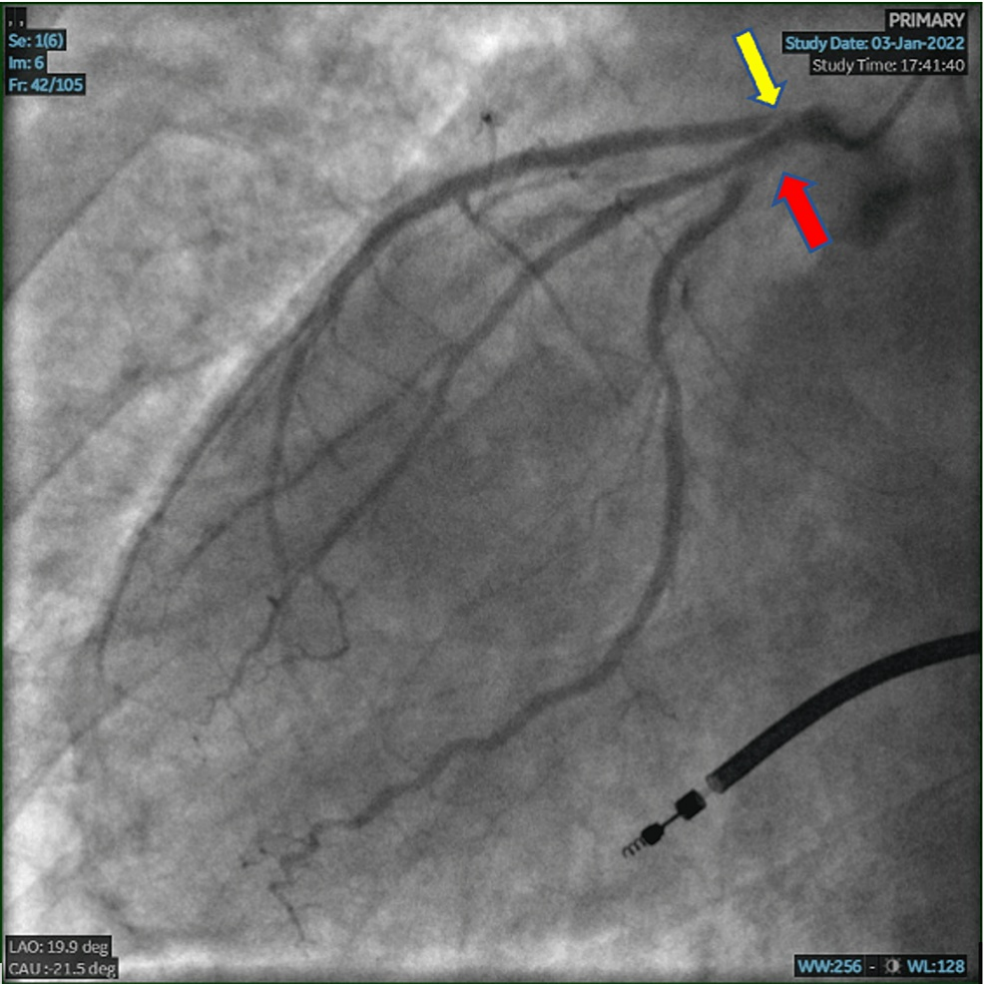
Left anterior oblique caudal view of left coronary system Ostium to proximal left anterior descending 99% stenosis (yellow arrow) and ostium to proximal left circumflex 90% stenosis (red arrow) are seen.

A transthoracic echocardiogram was performed showing an ejection fraction (EF) of 40% (prior 50%) with systolic function being moderately reduced, left atrium dilation, and new hypokinetic regions including mid anterior, mid inferoseptal, mid anterolateral, apical anterior, apical septal, apical lateral, and apex. Due to these findings as well as the patient’s symptoms, cardiac catheterization was performed. Results included: ostium LAD to proximal LAD 99% stenosed, ostium to proximal Circumflex 90% stenosed, Ramus 10% stenosis, distal right coronary artery (RCA) 50% stenosed, right posterior descending artery (RPDA) 70% stenosed, and mid RCA 30% stenosed (Figure 1). 

With these extensive catheterization findings, coronary artery bypass grafting (CABG) was recommended; however, it was not offered given patient requiring 6 L of oxygen in addition to extensive ground-glass opacities seen on CT imaging in the setting of COVID-19 pneumonia. The patient was then transferred to a different hospital for potential high-risk PCI. At this current time, the patient was started on Dexamethasone, Remdesivir, Baricitnib for his severe COVID-19 infection, nitroglycerin drip, aspirin, and switched from atenolol to metoprolol. Dofetilide was also continued from the patient's home medication regimen.

**Figure 2:**
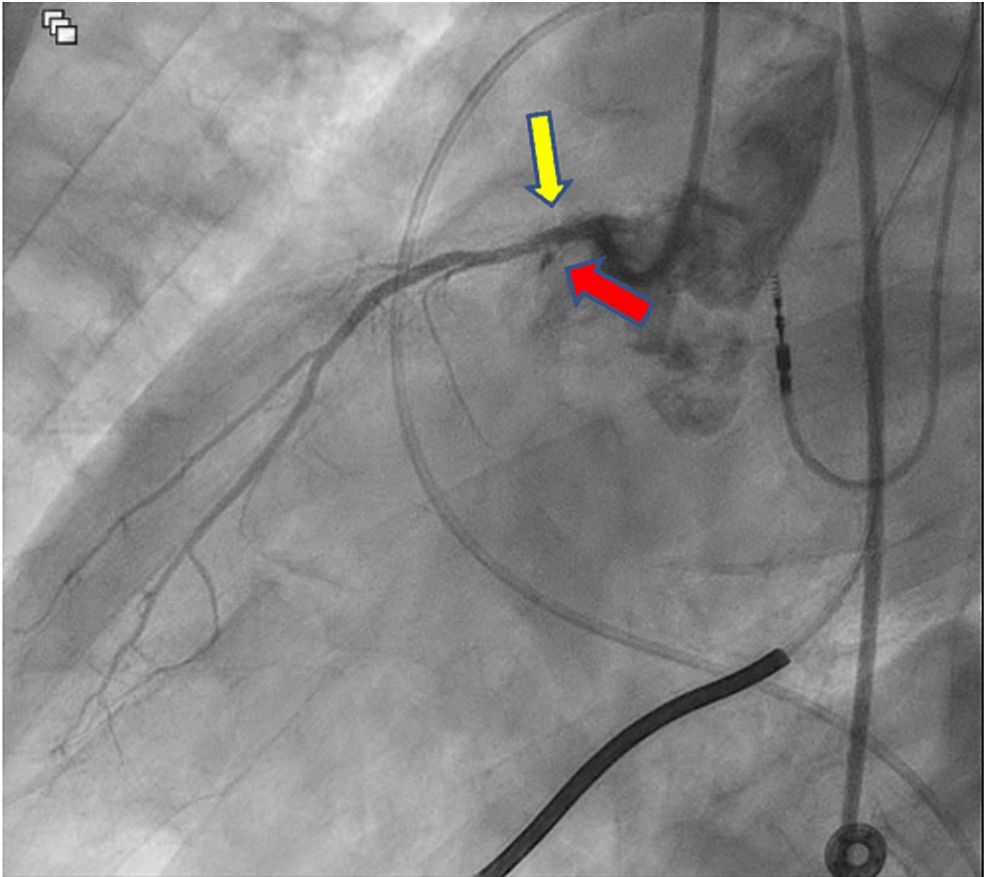
Left anterior oblique caudal view Ostium left anterior descending complete stenosis (yellow arrow) and ostium left circumflex 90% stenosis (red arrow) are seen.

**Figure 3:**
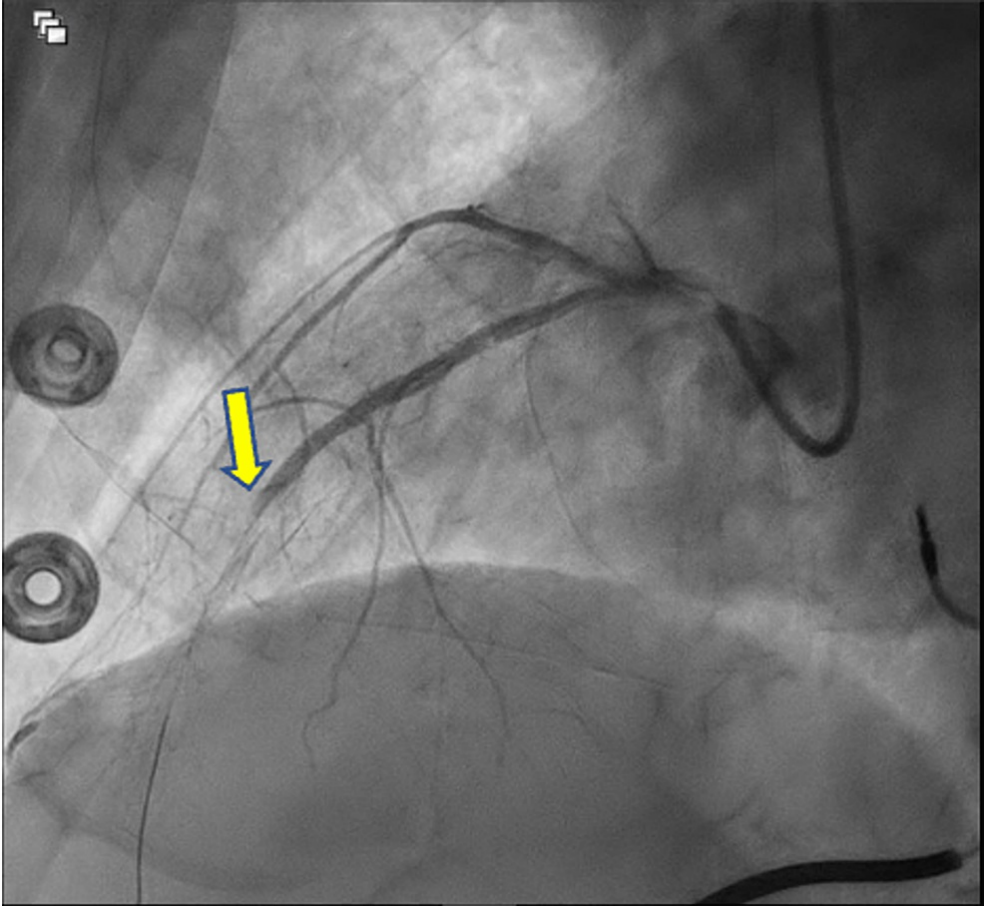
Left anterior oblique caudal view Mid-Left anterior descending 99% stenosis (yellow arrow), which was not amenable for percutaneous coronary intervention and was managed medically.

Upon arrival at a different hospital, the patient had an acute thrombotic episode manifesting as an anterior STEMI and he was immediately taken to the cardiac catheterization lab and had a successful salvage STEMI PCI with a 3.5 x 23 mm Xience Skypoint DES in the ostial LAD (Figure 2). The mid-LAD was occluded and unsuccessfully opened (Figure 3). Additionally, the patient had left circumflex disease (90% stenosed), but due to the complexity of the case and length of the procedure, stent placement was not attempted and managed medically. The patient became symptomatically better, and the COVID-19 pneumonia resolved. The patient was discharged on coumadin in the setting of left ventricular (LV) apical thrombus that was found on repeat echocardiogram as well as dual antiplatelet therapy (DAPT) with Prasugrel and aspirin with the intent to discontinue aspirin at one month.

One month follow-up transthoracic echocardiogram showed an EF of 45% with systolic function being mildly reduced. Moderate hypokinesis was seen of the basal to mid-anterior, anteroseptal, and inferoseptal walls. Severe hypokinesis of the apex, apical anterior, and apical septal walls were also seen. No LV thrombus was appreciated. Additionally, a cardiac MRI was performed for better imaging at two-month follow-up which showed mildly enlarged left ventricle size with mild to moderately reduced systolic function with an ejection fraction of 45%. Additionally, hypokinesis of the mid anteroseptal, inferoseptal, and apical anterior walls were noted consistent with ischemic cardiomyopathy.

When presenting to the outpatient office, the patient noted intermittent episodes of chest pain, shortness of breath, and palpitations although his symptoms were significantly improved. At a six-month follow-up visit, he was having significantly fewer symptomatic episodes. His ICD device readings showed occasional PVC’s and his device setting was changed to dual chamber pacing which significantly helped with his symptoms. At this visit, the decision was made to defer repeat cardiac catheterization for LCX occlusion and medically manage with addition of cardiac rehabilitation.

At his nine-month follow-up, the patient was still having few episodes of palpitations. The patient was exercising regularly and had a very high functional capacity, typically not seen in this specific patient population with significant disease burden. Holter monitor testing was offered for his palpitations, but the patient declined. Patient is continuing cardiac rehab with close follow-up with multidisciplinary teams.

## Discussion

Our patient presents with a complex presentation of COVID-19 infection with a favorable outcome despite a very high-risk clinical course. The most prevalent medical condition in patients with COVID-19 infection is a cardiovascular disease with a 73.5% association rate [2]. Among COVID-19 patients with cardiovascular disease, 8%-12% of patients were found to have an acute cardiac injury [4]. Additionally, a statistically significant increase in mortality (10.2% vs 5.2%) was seen in hospitalized COVID-19 patients with prior cardiac disease vs patients without the prior cardiac disease [5]. In this specific case, the patient has an extensive history of cardiac disease including atrial flutter, multiple coronary stent placements, coronary artery disease, hypertension, and prior history of cardiac arrest requiring ICD placement.

Patients presenting with COVID-19 and STEMI have been found to have higher rates of stent thrombosis (10.3% vs 1.2%), greater incidence of multiple thrombotic lesions, and higher thrombus grades [3]. Cardiogenic shock after PCI (9.9% vs. 3.8%) and mechanical thrombectomy (44% vs. 33.5%) were found to be significantly greater in COVID-19 patients vs. those without. These findings are consistent with our patient that despite past PCIs and medical management, he presented with extensive coronary artery disease.

LV thrombus is a rare complication of COVID-19. With this complication, the mortality rate is roughly 23% and STEMI diagnosed in 33% of patients with LV thrombus died [6]. Additionally, acute myocardial infarctions are found to be associated with thrombus formation [7]. Although an association has been found and cases have been reported, the number of cases is quite limited. Additionally, the cases found a trend toward milder COVID-19 such as a case of LV thrombus in the setting of dilated cardiomyopathy in a patient with mild disease [8]. Our specific case is unique in that our patient had severe COVID as seen on a CT scan as well as an active STEMI. This type of case with the presentation of LV thrombus has been extremely rare with very few cases reported.

Management of these patients can be very complex at times as seen in this specific patient requiring a multi-disciplinary approach. Some of the medications such as Baricitnib that are used to treat severe COVID may have thrombotic effects, which would be contraindicated in these patients. However, in our case, the patient's COVID and clinical status were so severe, that the benefits outweighed the risks. Overall, the cornerstone of management is revascularization. In our specific case, revascularization was very difficult due to the extent of coronary disease hence the patient being transferred to another facility. Revascularization was finally achieved in the patient’s LAD, but medical management had to be chosen for his LCX disease due to the complexity of the case.

At a follow-up visit, the patient was still experiencing symptoms including chest pain, shortness of breath, and palpitations. Multiple adjustments including threshold settings were made to the patient's ICD and cardiac rehabilitation was started. Given the significant improvement in his functional status and symptoms, cardiac rehabilitation with observation and medical management was chosen.

## Conclusions

COVID-19 has been known to increase thrombotic risk and presents unique decision-making and management challenges. This case demonstrates the management decisions through a thorough discussion of the clinical course as well as decision-making for this patient that is not routinely seen. Additionally, the finding of LV thrombus and management of this thrombus in addition to the STEMI highlights the increased attention that should be placed on these patients due to the pro-thrombotic state that occurs with COVID-19. Close outpatient monitoring, a multidisciplinary team approach, and prompt management of ACS are key to favorable outcomes in this patient population.
